# Methodological recommendations for assessing the impact of adaptations on outcomes in implementation research

**DOI:** 10.1186/s13012-025-01441-8

**Published:** 2025-06-23

**Authors:** Kelly A. Aschbrenner, Borsika A. Rabin, Stephen J. Bartels, Russell E. Glasgow

**Affiliations:** 1 Department of Psychiatry, Dartmouth Hitchcock Medical Center, One Medical Center Drive, Lebanon, NH 03756 USA; 2 UC San Diego, The Herbert Wertheim School of Public Health and Human Longevity Science, 9500 Gilman Dr, La Jolla, CA 92093 USA; 3 Altman Clinical and Translational Research Institute, Dissemination and Implementation Science Center, 9500 Gilman Dr, La Jolla, CA 92093 USA; 4 The Mongan Institute, Department of Medicine, Massachusetts General Hospital, 100 Cambridge Street Suite 1600, Boston, MA 02114 USA; 5 Harvard Medical School, 25 Shattuck Street, Boston, MA 02115 USA; 6 Department of Family Medicine and ACCORDS Dissemination and Implementation Science Program, University of Colorado Anschutz Medical Campus, 890 N Revere Ct, Aurora, CO 80045 USA

**Keywords:** Adaptations, Fidelity, Implementation outcomes, Study designs, Evidence-based interventions, Implementation strategies

## Abstract

**Background:**

A major gap in implementation research is guidance for designing studies to assess the impact of adaptations to interventions and implementation strategies. Many researchers regard experimental designs as the gold standard. However, the possible study designs for assessing the impact of adaptation on implementation, service and person-level outcomes is broad in scope, including descriptive and correlational research and variations of randomized controlled trials. This article provides a set of key methodological recommendations for assessing the impact of adaptations to interventions and implementation strategies on implementation outcomes.

**Recommendations:**

We offer four key recommendations for investigating the impact of adaptations on implementation outcomes. First, we recommend defining the construct of adaptations and identifying the type and timing of adaptations. Second, we recommend that study teams identify the expected proximal and distal outcomes of adaptations. Third, we recommend that study teams consider all possible study design options and select the design that is best suited to answer the research question(s), and is feasible given practical and technical constraints, and acceptable to research partners and participants. Fourth, we recommend that study teams consider the type of adaptation and outcome data available, the goals of the adaptation study, and the complexity of the study design when selecting analytic approaches. We provide materials and examples related to the four key recommendations to help study teams plan and conduct adaptation studies.

**Conclusions:**

This article provides methodological recommendations for assessing the impact of adaptations to interventions and implementation strategies on implementation, service, and person-level outcomes that are grounded in the practical realities of implementation research. Increasing the number of studies examining how, which, and under what conditions adaptations are associated with mechanisms and outcomes will advance research on adaptation.

**Supplementary Information:**

The online version contains supplementary material available at 10.1186/s13012-025-01441-8.

Contributions to the literature

- A major gap in the implementation science literature is methodological guidance for designing studies to investigate the impact of adaptations on relevant implementation outcomes.
- The methodological recommendations offered in this article focuses on investigating the impact of adaptations to interventions and implementation strategies.
- We articulate key recommendations for investigating the impact of adaptations on implementation, service and person-level outcomes; provide materials and examples to inform study design selection; and present a matrix for specifying adaptations and their anticipated proximal and distal outcomes.

## Background

The need to adapt both evidence-based interventions (EBIs) and implementation strategies to improve their fit with new populations and contexts has been well-established within the implementation science community [1, 2]. Adaptation has been defined as “a process of thoughtful and deliberate alteration to the design or delivery of an intervention, with the goal of improving its fit or effectiveness in a given context” [3]. EBIs have been adapted to enhance effectiveness and efficiency; improve feasibility, usability, scalability, or sustainability; and address inequities [4–6]. Researchers have also deliberately modified the existing context to better align with EBIs [7]. While the focus of most prior research has been on adaptations to EBIs, there has been an increasing emphasis on the need to also adapt implementation strategies to the context in which EBIs are implemented [8–12]. Despite compelling practical and theoretical reasons for adaptation, relatively little is known about the impact of adaptations on implementation, service, and person-level outcomes [13]. Consequently, there is little guidance on the types of adaptations and outcomes that should be the focus of studies to advance both implementation research and implementation practice.

Implementation science tools and frameworks are available to guide the systematic planning, tracking, and reporting of adaptations [3, 14–18]. For example, the ADAPT guidance proposes a process model for adapting and transferring EBIs to new contexts [18]. ADAPT provides a framework and step-by-step guidance for involving stakeholders throughout the adaptation process, piloting and evaluating an adapted intervention and if the intervention is feasible and effective, implementing and maintaining the adapted intervention at scale. When documentation and reporting of adaptations is of interest, the Framework for Reporting Adaptations and Modifications-Enhanced (FRAME) [3, 19] is widely used for systematically documenting the reasons, timing, context, and process of modifying interventions. Similarly, the Framework for Reporting Adaptations and Modifications to Evidence-based Implementation Strategies (FRAME-IS) [17] was one of the first reporting tools for documenting modifications to implementation strategies. Both the FRAME and FRAME-IS guide study teams in describing what is modified, the nature of the modification (e.g., tailoring, adding or removing elements), and the goal of the modification (e.g., increase reach). These frameworks provide the structure for classifying adaptations that is critical for proposing interrelationships among the process, types, and reasons for adaptations and implementation, service, and person-level outcomes.

The Model for Adaptation Design and Impact (MADI) [20] uses constructs from implementation science frameworks, including the FRAME [3], to guide study teams in creating an explanatory model for adaptations’ impact on outcomes. MADI was created to help practitioners and researchers think through the intended and unintended effects of adaptations on outcomes and investigate relationships among relevant constructs. Another useful model for conceptualizing the relationship between adaptations, potential moderators and mediators, and implementation outcomes is the Practical, Robust Implementation and Sustainability Model (PRISM). PRISM includes multilevel contextual domains and the Reach, Effectiveness, Adoption, Implementation, and Maintenance (RE-AIM) outcomes used to tailor iterative adaptations based on implementation priorities and progress on different outcomes [21–23].

In this article, we provide key methodological recommendations supported by materials and examples for assessing the impact of adaptations on implementation, service and person-level outcomes. Building on existing definitions, we conceptualize adaptations as any planned or unplanned change to the intervention or implementation strategy that occurs before, during, and after implementation*.* An adaptation study is a study of changes made to an intervention or an implementation strategy, including planned and unplanned adaptations. An adaptation study could be a primary stand-alone study (e.g., primary aim to study adaptations) or an add-on study to complement an existing study (e.g., secondary or tertiary aim to study adaptations). We recommend study teams, which includes researchers and their clinical and community partners, consider using the full range of applicable study designs, and select the design that is best suited to answer the research question while balancing the practical and technical constraints of the study, and ensuring that the design is acceptable to all study partners.

The four key recommendations outlined in this article include: 1) define the construct of adaptations and identify the type and timing of adaptations; 2) identify and specify the expected proximal and distal outcomes of adaptations; 3) consider all possible study design options and choose the design that is best suited to answer the research question(s) while balancing logistical constraints and challenges; and 4) consider the intersection between available data and the goals of the adaptation study when selecting the most appropriate analytic approaches. The four key recommendations are discussed below with supporting materials and case examples.

### Operationalize adaptations and report the type and timing of adaptations

To establish a relationship between an adaptation or a bundle of adaptations and outcome(s), it is critically important to specify both the adaptation(s) and the outcome(s). There are many characteristics of adaptations that could be considered. For example, FRAME [3] specifies ten different characteristics along with multitude of sub-categories within each characteristic. FRAME-IS [17] includes 16 characteristics organized under seven modules and distinguishes core and optional modules to fit a particular study. The goal of these frameworks is not for study teams to measure and track all of the adaptation-related domains, constructs, and characteristics. Instead, study teams should choose the specific constructs that are most relevant to their research question(s), considering factors such as significance, innovation and potential impact of the adaptation. We recommend that study teams determine early in the planning phase of an adaptation study which adaptation constructs will be documented and what level of documentation is feasible. When deciding which constructs to document or what constructs to include in analysis of adaptation impact, we recommend specifying those that are hypothesized to be related to the intervention or strategy outcomes.

When time and resources are limited, when assessments are conducted after the adaptations occurred, and/or when a large number of adaptations are made, we recommend that study teams assess seven key aspects of adaptations. These include 1) what specifically was adapted (e.g. which activities or components of the protocol); 2) the focus (e.g. the intervention or implementation strategies, or context); 3) the purpose of the adaptation (e.g. to enhance reach, improve equity, increase fidelity or consistency of delivery, enhance sustainability); 4) the timing and sequence of adaptations (e.g., time since initial delivery of the intervention or strategy); 5) whether the adaptation was bundled with other adaptations; 6) the scope (e.g., whether all participants are exposed to the adaptation) and frequency of exposure to adaptations; and 7) whether the adaptation was planned or made in response to emerging events, data, or other factors. Collecting information on when adaptations occurred can inform specifying the timing of the outcomes as proximal or distal.

We recommend that the frequency and method of tracking adaptations be defined by the research question and informed by what is feasible given practical and technical constraints, and acceptable and appropriate to research partners. The field of implementation science has generated numerous methods to track and assess adaptations to context, interventions and implementation strategies that can be used in adaptation studies that stand alone or are add-ons to existing studies [2, 24–28]. For some studies, it may be desirable and feasible to assess adaptations as close to real time as possible (e.g., documentation of adaptations during team meetings), while for other adaptation studies, including those that are add-ons to existing studies, it may be more appropriate to assess adaptations at the end of the implementation phase (e.g., retrospective assessment of quarterly notes). Whether adaptations are assessed throughout or at the end of a study, using multiple methods including interviews and observations that include the perspectives of different stakeholders may identify meaningful adaptations.

When a large number of adaptations have been documented, as is often the case, a decision should be made as to which ones to prioritize when investigating the impact of adaptation on outcomes. One approach to prioritizing adaptations is to distinguish between adaptations that impact the fixed aspects (i.e., essential functions) of an intervention or strategy vs. the variable aspects (i.e., form in different contexts [29, 30]. Adaptations should be prioritized that are expected to have a major impact on both the core functions or mechanisms and the implementation outcomes. For example, for a health promotion intervention it may be more important to examine the effect of an adaptation on the essential function of delivering supported exercise sessions, than an adaptation to where the exercise sessions take place (e.g., community-based fitness facility or health center). When making decisions about which adaptations to prioritize, we recommend that researchers include clinical and community partners in the decision making process to ensure the adaptations assessed meet the overall goal of improving the fit between the intervention or strategy and the setting and population.

### Identify the expected proximal and distal outcomes of adaptations

This section (and accompanying Tables 1 and 2 [along with Additional Files 1 A-B]) provides recommendations and a template that study teams can use to conceptualize, assess, and report both proximal and distal outcomes of adaptations to interventions and/or strategies. Table 1 (see Additional File 1 A for a blank template) has space to record information on factors related to adaptation impact. These include: 1) whether the outcome is an intended or unintended effect of adaptation; 2) whether or not the adaptation is equity-relevant; 3) timing of the anticipated outcome(s) as proximal (i.e., an immediate outcome) or distal (i.e., an outcome occurring at a later time); 4) the anticipated impact of the adaptation on mechanisms through which the intervention or implementation strategy works; and/or 5) implementation and person-level outcomes. If one of these constructs is not expected or relevant to your study, list ‘Not applicable.’ We anticipate that most cells in the table will not be completed and recommend that study teams complete the cells most relevant to the adaptation or adaptation bundle in their research. Ideally, this table should be completed close to the time the adaptation is made.

**Table 1:** Documenting and anticipating the impact of adaptations on proximal and distal outcomes

Type of Outcome	Equity-Relevant^a^	Type and Timing of Outcome					
		Proximal			Distal		
		Mechanism^b^	Implementation	Service/Person-level	Mechanism	Implementation	Service/Person-level
Example 1. Adaptation to Recruitment Materials: Tailored language for recruitment letter to increase cultural appropriateness for the priority community							
Intended	YES	Increased cultural appropriateness of the recruitment letter increases trust and interest from community members from the priority community	REACH: Increased number of community members from priority communities enroll in the study	N/A	N/A	REACH: More diverse sample reached across the study	N/A
Possible unintended—positive		N/A					
Possible unintended—negative		N/A					
Example 2. Intervention Adaptation: Increase support for providers delivering the intervention from clinic staff							
Intended	YES	Increase support for providers from clinic staff to deliver the intervention	IMPLEMENTATION: More consistent delivery of high quality intervention				Person-level: More patients experience positive outcomes
Possible unintended—positive		N/A					
Possible unintended—negative		Staff needs to take away time from patient follow-up calls for lab results	REACH: Less patients receive timely results from labs	N/A	N/A	N/A	SERVICE: Delay in care delivery for patients of the clinic

^a^Equity-relevant adaptations are defined as adaptations that explicitly aim to reduce health disparities by ensuring that interventions are accessible, acceptable, and effective for populations that experience disproportionate burdens of disease

^b^Mechanisms are underlying processes or drivers of the effects of interventions and strategies

**Table 2:** Specifying outcomes, data collection methods and timing of data analysis for investigating the impact of adaptations on outcomes in implementation research

Outcome	What data will be collected, from whom, when, and how about this impact/outcome?	How, when, and how frequently will this outcome be analyzed?
Example 1. Adaptation to Recruitment Materials: Tailored language for recruitment letter to increase cultural appropriateness for the priority community		
Reach: Increased number of community members from priority communities who enroll in the study. The timing of this outcome is considered proximal	Study registration records will be used to document recruitment including the number and key characteristics of the community members (e.g., primary language, race/ethnicity) who enroll in the study. The data will be collected throughout the study recruitment and enrollment period	The research team will conduct a weekly review of recruitment of priority community member characteristics by examining descriptive statistics (i.e., frequencies by characteristics) throughout the study recruitment and enrollment period
Reach: Diverse sample reached across the study. The timing of this outcome is considered distal	Participant data including key characteristics of the community members will be documented using study registration records. The research team will also document ongoing engagement in study activities both as numbers and participant characteristics throughout the entire study	At the end of the active implementation phase of the study, participant enrollment numbers and their characteristics will be reviewed as descriptive statistics (i.e., frequencies by characteristics). Data will also be reviewed longitudinally to explore critical windows when participants might have discontinued participation
Example 2. Intervention Adaptation: Increase support for providers delivering the intervention from clinic staff		
Implementation: More consistent delivery of high quality intervention. The timing of this outcome is considered proximal	A fidelity checklist completed by the providers and clinic staff will be used to document intervention delivery during implementation	Periodic (monthly) fidelity levels will be reviewed by the implementation team as the percentage of items delivered by providers and staff and what items were most or least likely to be delivered
Person-level: More patients experience positive outcomes. The timing of this outcome is considered distal	Effectiveness data will be collected from EHR that will include patient lab results and through interviews with patients assessing patient-reported outcomes of satisfaction with the intervention and quality of life. This data will be collected at the end of the implementation of the intervention	The quantitative data will be analyzed as pre- and post-adaptation. The qualitative data regarding satisfaction with outcomes and quality of life will be analyzed following implementation of the intervention
Possible unintended negative outcomes of Reach: Fewer patients receive timely results from labs. The timing of this outcome is considered proximal	The time from sample collection to results will be assessed using the EHR monthly throughout implementation	The data will be analyzed by calculating the time needed to deliver results from the lab and comparing the time for pre- and post-adaptation cases monthly throughout implementation
Possible unintended negative Service outcome: Delay in care delivery for patients of the clinic. The timing of this outcome is considered distal	The time from request for visit to appointment and from appointment to care delivery will be assessed using the EHR monthly throughout implementation. In addition, patient satisfaction with care delivery speed will be assessed via interviews at the end of the implementation of the intervention	The data will be analyzed by calculating the time to appointment and from appointment to care and comparing time for pre-and post-adaptation cases monthly throughout implementation. The qualitative data regarding patient satisfaction with care delivery speed will be analyzed following implementation of the intervention

It is important to strive to identify intended and unintended effects caused by an adaptation. Unintended effects caused by adaptations may have unexpected negative consequences or positive impacts on outcomes [20]. Unintended effects are difficult to anticipate but important to consider early in a study, especially those that might impact health equity. A positive unintended effect might indicate additional benefits on person-level outcomes, such as improved quality of life, whereas a negative unintended effect might increase inequities in access to care. Equity-relevant adaptations are defined as adaptations that explicitly aim to reduce health disparities by ensuring that interventions are accessible, acceptable, and effective for populations that experience disproportionate burdens of disease [31]. To center equity in the adaptation process, we recommend: 1) actively involving populations affected by inequities in the co-design and adaptation process to ensure interventions align with community strengths, values, and priorities; and 2) identifying and addressing health system barriers, policy-level influences, and provider-level biases that could hinder equitable implementation.

The study team should consider the timing of the anticipated effects(s) as proximal or distal and the projected impact on mechanisms through which the adaptation works. Proximal outcomes refer to immediate observable changes or effects of an adaptation, whereas distal outcomes refer to the long-term outcome of adaptations [32]. For each proximal and distal outcome, we recommend identifying the mechanism, defined as underlying processes or drivers of change [33], and examining if and to what extent adaptations impact underlying mechanisms driving the effects of interventions and strategies to ensure that core theoretical underpinnings are preserved. Finally, we recommend identifying implementation and service or person-level outcomes using either the Reach, Effectiveness, Adoption, Implementation, and Maintenance (RE-AIM) [34, 35] dimensions or Proctor et al. [13] categories of implementation outcomes.

Example 1 in Table 1 illustrates expected outcomes of an adaptation to an EBI recruitment strategy to make it more culturally appropriate for the priority community. Culturally tailored recruitment materials are materials designed to engage and encourage participation from diverse populations in research or clinical programs while affirming and respecting their cultural values, language, beliefs, and communication preferences [36]. In this case example, the study team received feedback from community members that culturally tailored recruitment letters were preferred over recruitment messages delivered via a patient portal. The primary intended effect of the adaptation is expected to be improved program reach indicated by increasing the number of participants from priority communities that enroll in the study. Distal outcomes include reaching a more diverse sample across the study. This adaptation is equity related as it is expected to increase the diversity of participants by adapting the content and style of the letter to the cultural characteristics of the priority community [37]. In this example, the research team was not able to identify any unintended impacts, positive or negative for this adaptation.

In Example 2, the study team was interested in assessing the impact of increasing support for supervising physicians delivering an intervention by increasing time and resources for other clinical staff to implement the intervention. This adaptation was expected to have a proximal impact on implementation quality and consistency and a distal impact on patient outcomes. These effects are hypothesized to be mediated by additional time and staff perceptions of support and priority of this intervention. The team identified a potential negative unintended consequence of this adaptation being delayed reporting of patient lab results due to the mechanism of reduced time for staff to conduct follow-up calls with lab results. The delayed reporting of patient lab results increases the risk of delayed diagnosis and treatment.

Once the study team has identified anticipated impacts related to each adaptation, the team should then determine what data will be collected, from whom, when, and how and the plan for evaluating each outcome. Table 2 provides a summary for each previously introduced example and Additional File 1B provides a template for study teams to enter this information for their own projects. When available and appropriate, data on outcomes may be collected from the Electronic Health Records (EHR). Some outcomes may be best captured via quantitative methods while others by qualitative methods and some through mixed methods involving the collection and integration of both quantitative and qualitative data.

For Example 1, the primary data type needed to evaluate the impact of adaptations are the enrollment records for the study collected by the study team. The same data source can inform impact for both proximal and distal reach outcomes. For example, whether a change in recruitment approaches led to a more diverse sample includes data on the initial recruitment of participants from the priority community and whether the overall study sample was more diverse. In addition, data about participants’ membership in the priority community (assessed by primary language and/or race and ethnicity) can be collected in the study registration database.

For Example 2, there are multiple outcomes including proximal implementation, distal person-level, and possible unintended negative proximal reach and distal service impacts. The timing and type of data to assess potential impacts will be different across the various outcomes. For example, different time points and sources of data collection can facilitate determining if an adaptation designed to increase support to clinical providers led to the unintended negative impact of delay in care delivery. Key outcomes include assessing the time needed to get an appointment and receive care and comparing care delivery speed for pre-and post-adaptation cases using data from the EHR and interviewing patients about their satisfaction with care delivery speed.

In summary, it can be challenging to specify the types and timing of adaptation impacts, and especially unintended impacts in advance. However, completing Tables 1 and 2 in Additional Files 1 A-B will help study teams make decisions about how and when to assess both adaptations and outcomes, and to make specific hypotheses about impacts. This is especially important when there are multiple adaptations and numerous types of outcomes being studied.

### Choose the design that is best suited and most feasible to answer the adaptation-related research question(s)

We recommend that study teams select a study design that is best configured and most feasible to investigate the impact of adaptations on outcomes of interest. There are many different study designs available for investigating adaptation impact, ranging from descriptive and correlational research used to explain the relationship between adaptations and outcomes without making any claims about cause-and-effect to experimental studies that examine the effect of the adapted intervention or implementation strategy on outcomes [38–40]. Study designs commonly used in implementation research include experimental, quasi-experimental, observational, and hybrid effectiveness-implementation studies [41]. In a review of study protocols used to test the effectiveness of implementation strategies, Mazzuca and colleagues reported that the majority (77%) of the studies in the sample (*N* = 404) used randomized designs; however, the authors noted that the use of alternative designs (e.g., adaptive designs) increased over time [42]. Hamilton and Finley [43] underscored the value of using qualitative research during implementation studies as a tool for investigating how and why implementation efforts succeed or fail. Furthermore, Palinkas and colleagues [44] emphasized the utility of mixed methods study designs where both quantitative and qualitative data is collected, analyzed and integrated to evaluate and/or understand implementation processes and outcomes. Each of these designs can be used to assess the impact of adaptations on outcomes. Additional File 2 provides a comprehensive list of study design options for assessing the impact of adaptations and examples for each type of study design.

**Fig. 1:**
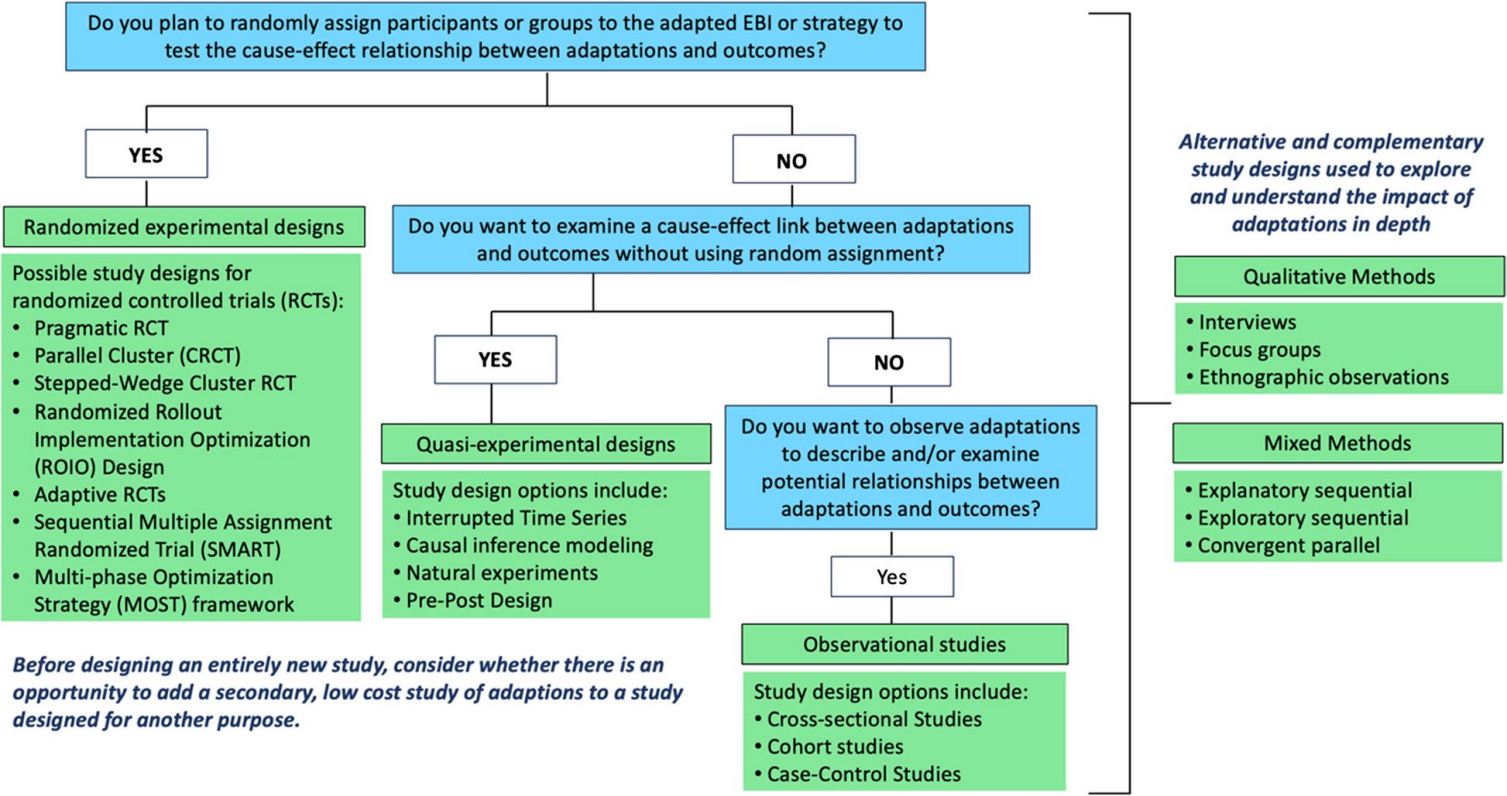
Decision tree for selecting a study design to assess the impact of adaptations

We recommend that study teams consider all possible study design options and choose the design best suited to answer the research question(s), most feasible given the practical and technical constraints of the study, and acceptable to research partners and study participants. An adaptation study could be a primary stand-alone study or an add-on study to complement an existing study. We have developed a diagram (Fig. 1) to guide study teams toward selecting a study design to assess the impact of adaptations on outcomes in implementation research. The decision diagram starts by asking whether the study team plans to (or has) randomly assign participants or groups to receive an adapted intervention or implementation strategy in order to test the cause-effect relationship between adaptations and outcomes. If yes, then a randomized study design would be the design of choice. If not, then the study team is asked whether they want to examine a cause-effect link between adaptations and outcomes without using random assignment. If yes, then quasi-experimental designs may be the design of choice. If not, then the study team is asked if they want to observe adaptations in order to describe and/or examine relationships between adaptations and outcomes. If yes, then observational studies may be the design of choice. We also highlight qualitative and mixed methods study designs as alternative and/or complementary approaches to understanding the impact of adaptations on outcomes in greater depth.

Importantly, we encourage study teams to consider opportunities to embed low cost studies of adaptation when a study has been designed for another purpose. Whether the study design is a new experimental, quasi-experimental or observational study, or an add on to an existing study, understanding the impact of both planned and unplanned adaptations that occur during implementation in diverse contexts can inform continued learning and problem solving and generate hypothesis for future rigorous research on the impact of adaptations and outcomes.

### Consider the type of adaptation and data available, the goals of the adaptation study and the level of complexity of the study design when selecting analytic approaches

As Rabin, Cain and Glasgow previously stated [45], most adaptation studies have used descriptive statistics and qualitative methods to analyze data about adaptations. Descriptive research has been useful for reporting and characterizing adaptations without testing hypotheses about the relationship between adaptations and outcomes of interest. When the goal is to assess the impact of adaptations on outcomes, study teams should use analytic approaches that include inferential statistics that allow for hypotheses testing and estimation to make comparisons and predictions and draw conclusions about the relationship between one or more independent variables and the dependent variable [46].

Examples of analytic approaches that use inferential statistics to examine the impact of adaptations on outcomes have included regression models and Multivariate Analysis of Variance (MANOVA). Regression analysis is used to quantify how one variable changes with respect to another variable. Aschbrenner and colleagues [47] used a mixed effects logistic regression model to examine the association between the type of adaptations (i.e., fidelity-consistent vs. fidelity-inconsistent) and person-level outcomes (i.e., cardiovascular risk reduction defined as achieving clinically significant weight loss of > 5% body weight or clinically significant increase in cardiorespiratory fitness of > 50 m on the 6-Minute Walk Test) in a study of stakeholder-led adaptations to an evidence-based health promotion intervention. Parekh and colleagues [48] used logistic regression to examine outcomes (e.g., knowledge) of an evidence-based sexual health program in school settings by level of adaptation (low, middle, and high-adaptation). Schinckus and colleagues [49] examined associations between adaptations to a protocol for a diabetes self-management program and patient-level outcomes (e.g., self-care behaviors, diabetes specific health literacy) through logistic regressions and repeated measures MANOVA, a statistical technique that compares groups when there are multiple dependent variables.

Another rigorous analytic approach for assessing the impact of adaptations on implementation outcomes is Configurational Comparative Methods (CCM), a systematic approach for comparing cases to identify configurations of conditions that lead to a specific outcome [50, 51]. Coury and colleagues [51] used CCM to examine the combinations of adaptations to implementing mailed fecal immunochemical testing (FIT) for colorectal screening outreach and health system characteristics that together distinguished the health systems with higher screening rates from those with lower screening rates. Holtrop and colleagues [50] used a configurational technique to identify ways that adaptation components cluster together in unique patterns producing adaptation “types” that can be analyzed with outcome data to determine if the adaptations produce more favorable outcomes than adaptation components individually.

In developing a statistical analysis plan for an adaptation study, we recommend that study teams collaborate with their methodologist and statistician colleagues to design a statistical plan for their respective projects that considers the scope (i.e., participant exposure to the adaptation) and frequency of exposure to adaptations and how potential mediators are impacted by adaptations. In particular, content-related adaptations can differ in terms of scope and frequency of exposure, including whether all participants were exposed to the adaptation across every encounter (e.g., multi-session intervention) or whether adaptations were only applied for selected participants and/or during selected encounters. We also emphasize the need for study teams to include in their statistical analysis plans an examination of whether potential moderators that adaptations are intended to address (e.g., literacy, comorbidities, access barriers) are associated with proximal and distal outcomes. If so, we encourage study teams to consider whether the wrong adaptation was chosen, the adaptation was insufficiently applied, or other unaddressed structural/contextual factors continued to contribute to disparities in outcome.

We recommend that study teams consider the type of adaptation and outcome data available, the goals of the adaptation study and the level of complexity of the study design when selecting analytic approaches. Flexibility in the choice of an analytic approach may depend on whether the adaptation study is a primary stand-alone study or an add-on study to complement an existing study. Additional File 2 provides examples of studies with various analytic approaches that could be useful for assessing the impact of adaptations on outcomes.

## Summary

We build on the existing literature to offer practical methodological recommendations for assessing the associations and impacts of adaptations in implementation research. Four key recommendations outlined in this article include: 1) define the construct of adaptations and identifying the type and timing of adaptations; 2) identify and specify the expected proximal and distal outcomes of adaptations; 3) consider all possible study design options and choose the design that is best suited to answer the research question(s); and 4) consider the type of adaptation and outcome data available, the goals of the adaptation study and the level of complexity of the study design when selecting analytic approaches. The recommendations and accompanying materials provide practical guidance and tools for assessing the impact of adaptations and implementation strategies grounded in the realities of implementation research. Growing the number of investigations examining how and which adaptations are associated with outcomes of interest will advance research on adaptations.

## Supplementary Information

Additional file 1.

Additional file 2.

## Data Availability

Not applicable.

## Abbreviations

CRT: Parallel Cluster RCT
EBI: Evidence-based interventions
EHR: Electronic Health Records
FRAME: The Framework for Reporting Adaptations and Modifications-Enhanced
FRAME-IS: The Framework for Reporting Adaptations and Modifications to Evidence-based Implementation Strategies
MADI: Model for Adaptation Design and Impact
MANOVA: Multivariate Analysis of Variance
MOST: Multi-phase Optimization Strategy
NEs: Natural Experiments
PRISM: Practical, Robust Implementation and Sustainability Model
RE-AIM: Reach, Effectiveness, Adoption, Implementation, and Maintenance
RCT: Randomized Controlled Trial
ROIO: Randomized Rollout Implementation Optimization
SW-CRT: Stepped-Wedge Cluster RCT
SMART: Sequential Multiple Assignment Randomized Trial
ITS: Interrupted Time Series
