# Supplementary material for: Methodological recommendations for assessing the impact of adaptations on outcomes in implementation research
Source: Implement Sci. 2025 Jun 23;20:30. doi: 10.1186/s13012-025-01441-8 (PMC12183851; doi:10.1186/s13012-025-01441-8)
Supplement: Supplementary file 1 — Additional file 1. [file 13012_2025_1441_MOESM1_ESM.docx]

**Additional File 1A: Anticipating and documenting the impact of adaptations on proximal and distal outcomes**

| **Type of Outcome** | **Equity-**  **relevant^a^** | **Type and Timing of Outcome** | | | | | |
| --- | --- | --- | --- | --- | --- | --- | --- |
|  |  | **Proximal** | | | **Distal** | | |
|  |  | **Mechanism^b^** | **Implementation** | **Service/Person-level** | **Mechanism** | **Implementation** | **Service/Person-level** |
| **Adaptation brief description 1: (Purpose; action; who adapated what when):** | | | | | | | |
| **Intended** |  |  |  |  |  |  |  |
| **Possible unintended - positive** |  |  |  |  |  |  |  |
| **Possible unintended - negative** |  |  |  |  |  |  |  |
| **Adaptation brief description 2: (Purpose; action; who adapated what when):** | | | | | | | |
| **Intended** |  |  |  |  |  |  |  |
| **Possible unintended - positive** |  |  |  |  |  |  |  |
| **Possible unintended - negative** |  |  |  |  |  |  |  |

^a^ Equity-relevant adaptations are defined as **adaptations that explicitly aim to reduce health disparities by ensuring that interventions are accessible, acceptable, and effective for populations that experience disproportionate burdens of disease.**

^b^ Mechanisms are underlying processes or drivers of the effects of interventions and strategies.

**Additional File 1B: Assessing the impact of adaptations**

| **Outcome/Impact** | **What data will be collected, from whom, when, and how about this impact/outcome?** | **How, when, and how frequently will this outcome/impact be analyzed?** |
| --- | --- | --- |
| **Adaptation brief description 1** | | |
| Impact 1 from Additinoal File 1A |  |  |
| Impact 2 from Additional File 1A |  |  |
| **Adaptation brief description 2** | | |
| Impact 1 from additional File 1A |  |  |
| Impact 2 from Additional File 1A |  |  |
