# Supplementary material for: Methodological recommendations for assessing the impact of adaptations on outcomes in implementation research
Source: Implement Sci. 2025 Jun 23;20:30. doi: 10.1186/s13012-025-01441-8 (PMC12183851; doi:10.1186/s13012-025-01441-8)
Supplement: Supplementary file 2 — Additional file 2. [file 13012_2025_1441_MOESM2_ESM.docx]

Additional File 2. Study design options for assessing adaptation impact

| **Study Design** | **Advantages** | **Key Considerations and Challenges** | | **Examples** | |
| --- | --- | --- | --- | --- | --- |
| ***Experimental Studies:*** *Studies in which a study team deliberately introduces an intervention to observe its effects^1^.* | | | | | |
| **Randomized Controlled Trials (RCT).** RCT is a study design used to determine the causal effect of an intervention and/or implementation strategy on pre- determined outcomes. Participants or groups are assigned to receive the intervention (or strategy) or a comparator condition by a random process such as the generation of a random sequence of numbers.^1,2^ | - RCTs can be used to test the effect of planned adaptations to an intervention and/or an implementation strategy. - The use of a randomized design attempts to maximize internal validity (or the ability to draw causal inference) by balancing measured and unmeasured differences between experimental and a comparator to enhance the ability to determine the effect of an adapted intervention or strategy on outcome(s). - Comparators used in health related behavioral interventions include: a) no treatment or waitlist (often referred to as control condition); and b) usual care; optimized standard of care; alternative intervention; and alternative modality (often referred to as comparison condition).^3^ | - In many RCT designs, adaptations will need to be planned a priori and remain static in the RCT. - Adaptations are often tested as a whole package; performance of individual adapted components may be unknown. - If using a parallel RCT design, there may be resistance to or ethical issues to take into consideration when delivering adapted interventions and/or strategies to one experimental condition and not the comparator condition. Wait-list control RCTs, in which the intervention or strategy is delivered to those in the comparator condition after the trial has formally ended, may help mitigate some of these hesitations. | | **Intervention:** Researchers partnered with patients, families, and providers to plan adaptations to an evidence-based intervention guided by an implementation science framework. The study team then conducted an RCT to evaluate the effectiveness of the adapted intervention on patient-level health outcomes compared to usual care. This example of an RCT used to study the impact of adaptations is based on a study by Rosas and colleagues.^4^ | |
| ***Types of RCTs:*** *Pragmatic RCTs; Parallel Cluster RCTs; Stepped-Wedge Cluster RCTs; Randomized Rollout Implementation Optimization (ROIO) Design* | | | | | |
| **Pragmatic RCTs.** A type of RCT that is designed with the purpose and intent of answering questions that are of practical utility to partners and can generalize to the conditions under which the intervention and/or strategy would be received in usual care. Pragmatic RCTs are designed in a way to maximize external validity and enhance generalizability while maintaining strong internal validity through randomization to condition.^5-7^ | - Pragmatic RCTs allow for flexibility in the delivery of the intervention or strategy similar to the flexibility in usual care. - Participants in the trial should be similar to patients who would receive the intervention if it were part of usual care. - Clinicians delivering the intervention should be similar to the clinicians who would deliver the intervention or receive the implementation strategy in usual care. - The RCT setting is similar to usual care settings where an adapted intervention or strategy would be implemented. | - A pragmatic RCT often investigates a general approach to treatment rather than focusing on high fidelity to intervention or strategy delivery which can be both an advantage and a challenge when assessing the impact of planned adaptations on outcomes. Poor delivery (low fidelity) of an adapted intervention or strategy can negatively impact trial validity. - Follow-up data collection should be unobtrusive, often relying on electronic health records. This may limit a study team’s ability to collect multi-level contextual and process data that can inform an understanding of the impact of adaptations on outcomes. | | **Implementation Strategy:** Researchers conducted a pragmatic RCT comparing commonly used quality improvement strategies for supporting the implementation of an intervention in routine clinical practice settings. The trial was conducted at community health centers that shared a single, fully integrated, centrally hosted electronic health record. In addition, qualitative data collected and analyzed during the trial informed real time adaptations of the implementation support strategies. The outcomes of interest were clinical level results, categorized using the Reach, Effectiveness, Adoption, Implementation, Maintenance (RE-AIM) framework. This example of a pragmatic RCT is based on a study by Gold and colleagues.^8^ | |
| **Parallel Cluster RCT (CRTs)**. In CRTs, groups or clusters (e.g., hospitals, clinics, public health organizations) are randomized to study conditions, and observations are taken on the members of those groups (e.g., leaders, providers, patients, clients) with no-cross over of groups or clusters to a different condition or study arm during the trial.^9,10^ | - CRTs are an ideal comparative design when the study team wants to evaluate an adapted intervention and/or strategy that operates at a group level; manipulates or adapts the context (e.g., social or physical environment); or cannot be delivered to individual participants without substantial risk of between-group contamination. | - When the number of groups available for randomization in a CRT is limited (small), there is a greater risk that potentially confounding variables will be unevenly distributed among the study conditions, which can threaten internal validity of the trial. | | **Implementation Strategy:** Researchers planned adaptations to the way an implementation strategy was delivered. The research team then conducted a CRT in a large sample of behavioral health organizations to evaluate the impact of the adapted implementation strategy compared to a comparison condition on patient-level and implementation outcomes. This example of a CRT is based on a study by Bartels and colleagues.^11^ | |
| **Stepped-Wedge Cluster RCT (SW-CRT).** The SW-CRT is a study design that involves randomly assigned and sequential crossover of groups of clusters at regular intervals (“steps”) from an initial control period to the intervention until all clusters are exposed.^12^ | - All clusters eventually receive the adapted intervention or strategy. - Each cluster contributes observations under both control and intervention or strategy periods. - SW-CRTs do not implement the adapted intervention or strategy at all sites at once as staggered implementation can happen over time (logistical convenience). | - Threats to internal validity include fidelity losses to adapted intervention or strategy, particularly if adaptations are not guided and monitored throughout the trial. - Data collection burden can be high if collecting data on adaptations cannot be automated or use existing resources. - Some clusters may not be able to follow the randomization schedule which may pose logistical challenges. | | **Intervention:** Researchers conducted a SW-CRT of an adapted intervention. The study involved nine teams randomly allocated (3 at a time) to different start dates 9 months apart. Data were collected at the cluster (clinics) and individual levels (service users) at baseline before randomization and at 9, 18, and 27 months post-randomization. Study results showed no statistically significant differences in individual-level outcomes between the intervention and the control arms. The study team concluded that additional adaptations to the intervention may be required to increase the impact of the intervention. This example of an SW-CRT is based on a study by Palmer and colleagues.^13^ | |
| **Randomized Rollout Implementation Optimization Design (ROIO).** The ROIO is the same design as the SW-CRT where clusters of units (e.g., clinics) begin in a control group and then cross over into the intervention group at different time points and then continue with the intervention until the end of the study. However, in contrast to a SW-CRT, a ROIO design explicitly aims for iterative improvement in the intervention and/or implementation strategy between steps by adapting the strategy between clusters at each successive rollout.^14,15^ | - Builds upon the SW-CRT design in which all settings receive the implementation strategy by adding the feature that in later units the implementation strategy(ies) are purposely intended to be improved based on results from prior waves. - Potential to be highly acceptable to partners because each cluster will receive the intervention or strategy and the design allows for iterative refinement to improve the implementation strategies after each rollout. | - As with SW-CRTs, ROIO require repeated assessment of outcomes at different levels across the trial period, making these designs most suited for outcomes that can be assessed using routinely collected data or frequently collected data. | | **Implementation Strategy:** Researchers used a ROIO to implement and evaluate a multi-level and multi-component implementation strategy bundle that included strategies prioritized by community partners. Refinements to the implementation strategy bundle were made prior to each new roll-out based on information collected from the prior roll-out. Quantitative data were used to measure the impact of the implementation strategy bundle while qualitative data were used to summarize implementation experiences and identify necessary adaptations to the bundle prior to subsequent roll-outs. This example is based on a ROIO study protocol published by Stadnick and colleagues.^16^ | |
| ***Types of RCTs:*** *Adaptive research designs allow study teams to assess the effectiveness of individual intervention and implementation strategy components at prespecified interim time points during a trial and adapt interventions and strategies based on these observations*.^17^ | | | | | |
| **Adaptive RCTs.** In adaptive RCTs, outcomes are observed and analyzed at prespecified interim time points and adaptations to interventions and implementation strategies can be made based on these observations.^17,18^ | - Pre-specified decision rules dictate whether, how, and when to adapt the dosage, type, or delivery of interventions or strategies. - Participant characteristics (including prior response to treatment) are used in making decisions about adapted interventions or strategies. - The intervention or strategy is adaptive or dynamic or tailored because it responds to a person’s evolving characteristics. | - Some interventions or strategies are less ideal for sequential testing and some interventions or strategies cannot be applied sequentially. - In some cases, an initial ‘failure’ makes it highly unlikely the setting will engage in any later adaptations. - There may be insufficient empirical or theoretical basis for planning adaptations. | | **Intervention:** Researchers conducted a two-stage adaptive intervention trial in which all participants received an initial phase of treatment. At the end of the treatment period, proximal outcomes were assessed. Complete treatment responders continued with the original treatment while incomplete treatment responders received an augmented version of the original treatment. This example of an adaptive RCT is based on a study by Davis and colleagues.^19^ | |
| **Sequential Multiple Assignment Randomized Trial (SMART).** Sequential Multiple Assignment Randomized Trial (SMART) is a randomized experimental design used to build optimal time varying adaptive interventions whereby treatment is adapted and readapted over time in response to the specific needs of an individual or setting.^20-22^ The SMART multi-stage design enables researchers to modify the dose, type, or delivery of interventions and strategies based on prespecified decision rules. Participants are randomized to different interventions or strategies at each stage depending on response to previous stage. | - SMART designs allow study teams to assess the effect of adaptive approaches and to examine specific adaptations to interventions and/or strategies. - Useful when there is insufficient evidence or theoretical basis to assemble an adaptive intervention or strategy. - Enables researchers to take into account the order in which adaptations are presented rather than considering each adapted component in isolation. | - Key considerations in SMART include the need to decide how to assess early response/non-response and what criterion or cut-off should be used, what measure to use to determine response/non-response, and whether the measurement would be feasible to use in real world practice settings. | | **Implementation Strategy:** Researchers conducted a cluster randomized SMART trial comparing adaptive implementation strategies that begin with a standard or enhanced implementation strategy to improve EBP use and patient outcomes. Community-based outpatient clinics that did not respond to an evidence-based implementation strategy were randomized to receive additional support that included expertise in adapting the EBP in routine practice. This study will help determine the added value of more intensive implementation strategies within sites that need additional support to promote the uptake of EBP. This example is based on a SMART trial conducted by Kilbourne and colleagues.^23^ | |
| **Multi-phase Optimization Strategy (MOST).** MOST is a framework that incorporates the standard RCT, but before the RCT is undertaken MOST includes a method for identifying which components are active in an intervention or strategy, and which doses of each component lead to the best outcomes. After selecting active components of an intervention/strategy and identifying optimal doses in the screening and refining phases of MOST, respectively, the optimized intervention and/or strategy package is evaluated in an RCT.^20,24^ | - Applied with the objective of planning and optimizing an intervention or strategy before an RCT, often involves adaptation. - Applied to adapt intervention or strategy to local context by setting optimization objectives (e.g. identifying active components, optimal dose and most cost-effective combination of factors) tailored to context. | - Each adapted intervention or strategy component may have a small effect size and therefore, may limit the ability to draw valid inferences based on individual components of an intervention or strategy. - Results of the optimization phase may not generalize to other settings because of differences in optimization objectives. | | **Implementation Strategy:** A research team developed a study protocol to use the MOST framework to optimize and evaluate delivery strategies for a complex, multi-component evidence-based intervention. The team plans to use a multifactorial design to conduct a randomized factorial experiment of specific intervention delivery components. The team will estimate the impact of the delivery strategies on service and implementation outcomes, and perform exploratory analyses regarding interactions between delivery conditions and interactions with patient characteristics. The team will then evaluate the data to develop an optimized model of intervention delivery. This example is based on a study protocol by Broder-Fingert and colleagues.^25^ | |
| **Quasi-Experimental Studies:** *Quasi-experimental designs test causal hypothesis without random assignment to an intervention by using a comparison group or time period that reflect a counter-factual (i.e., outcomes if the intervention had not been implemented).*^2^ | | | | | |
| **Interrupted Time Series (ITS) Design.** An ITS design involves a collection of outcome data at multiple time points before and after an intervention/strategy is introduced at a given time point at multiple sites. The pre-intervention and/or strategy outcome data are used to establish a trend (i.e., counterfactual scenario) to which any change in outcome level or trend in the post-intervention period is attributed to the intervention/strategy.^26^ | - Advantage of an ITS design is that it can use data that are routinely collected on intervention or strategy adaptations with limited research burden. | - ITS require a clear differentiation of the pre-intervention period and the post-intervention period. - Requires a large number of observations both before and after an adaptation to permit assessment of the level and slope of the outcome data. - ITS works best with short-term or proximal outcomes that are expected to change relatively quickly after an intervention/strategy is implemented or after a defined lag. | | **Intervention:** Researchers used an ITS design to evaluate individual-level outcomes following the introduction of an adapted evidence-based intervention. The approach estimated what the outcomes would have been without the adapted intervention during a multi-year follow-up. The projection was based on the pattern of outcomes during a multi-year baseline period before the intervention was introduced. The difference between actual and projected outcomes provided an estimate of the impact of the adapted intervention. This example of an ITS is based on a study by Bloom and colleagues.^27^ | |
| **Natural experiments (NEs).** In NEs, the researcher does not control assignment to an intervention/strategy. NEs leverage the naturally occurring variation in exposure to estimate the impact of an event on outcomes of interest. NEs are often used as a study design when purposeful experimentation by the research team is not possible, such as when an exposure of interest cannot be practically or ethically assigned to research subjects.^28^ | - Ability to evaluate the introduction of interventions or strategies as they occur under real-world conditions. - When data already exist to evaluate a NE, researchers can generate timely evidence about the impact of an intervention or strategy. - Knowledge of the assignment process enables confounding due to selective exposure to be addressed. | - A key consideration in NE are the sources of variation in exposure and the expected effects. Intervention or strategies that are introduced abruptly, that affect large populations, and that are implemented where it is difficult for individuals to manipulate their exposure status are optimal for evaluation. - More complicated NE research designs may be needed where effects are smaller or more gradual. - Bias due to unmeasured confounding is a concern in NEs because of selective exposure to the intervention or strategy, although use of an instrumental variable can help mitigate some concerns. - Identifying adequate controls (unexposed groups) may be challenging. | | **Implementation:** Researchers evaluated the impact of changes to an implementations strategy for school-based body mass index screening on weight status for public school students. The natural experiment involved all school districts in a large state conducting annual BMI screening with optional parental notification of screening results. While rates of BMI screening increased over time, parental notification had no impact on subsequent BMI scores. The authors concluded that parental notification in its current form needed to be improved to have an impact on pediatric obesity. This example of a natural experiment is based on a study by Madsen.^29^ | |
| **Pre-Post Design.** Data are collected before an intervention and/or strategy is introduced (“pre” or baseline), and then compared to data collected after exposure to the intervention or strategy (“post” or outcome). A control or comparison group can be added that are not exposed to the intervention or strategy.^30^ Versions of the pre-post design include comparisons before-and-after intervention or strategy exposure (i.e., pre-post within group) or comparison between groups if a control or comparison group is included (i.e., pre-post between groups). | - Strength of temporality (before-and-after) to be able to suggest the outcome is influenced by the adaptation. - Especially useful for pilot testing an adapted intervention or strategy to identify areas that need refining prior to a larger-scale study. - Useful design when there are large-scale adaptations (e.g., changes to regulations or policies occurring across different settings). - Used when randomization not possible. | - Lack of control over elements that are changing at the same time as the adapted intervention or strategy is being implemented in a pre-post design without a control condition. - Value increased when there are multiple use cases in each category and results are replicated across settings. | | **Intervention and Implementation Strategy:** Researchers adapted an in-person intervention to be delivered remotely and added instructional coaching for providers implementing the intervention. The research team assessed the feasibility and acceptability of the intervention and implementation strategy and participant behaviors in a single arm pre-post study. The research team and their community partners used the results to make refinements to the intervention and implementation strategy prior to conducting a randomized pilot trial. This example of a pre-post design is based on a study by Wu and colleagues.^31^ | |
| ***Observational Studies:*** *A typed of study in which individuals are observed or outcomes are measured and no attempt is made by researchers to affect the outcome.^32^* | | | | | |
| **Cross-sectional Studies**. Cross-sectional studies are observational studies that analyze data from a population at a single point in time on both exposure and outcome.^33^ | - Cross-sectional studies can be used to describe planned and unplanned adaptations made to an intervention and/or strategy. - Can be quickly and efficiently added to an existing study to document and characterize adaptations. - Allows researchers to examine associations between adaptations and outcomes that can then be more rigorously tested in another design. - Provides information about the relationship between fidelity and adaptation that can be useful for future implementation research and practice. | - Cross-sectional studies do not distinguish between cause and effect. They cannot be used to determine if an adaptation causes an outcome, only if the adaptation is associated with outcomes. - Confounding may occur in analytical cross-sectional studies when another variable is associated with the exposure (i.e., adaptation) and influences the outcome. | | **Intervention:** Researchers measured and evaluated adaptations in the maintenance phase of intervention implementation. The research team rated adaptations as fidelity-consistent or fidelity-inconsistent. Study results showed that a greater number of adaptations were associated with significantly higher odds of positive health outcomes. With respect to the type of adaptation, the researchers found a significant positive association between the number of fidelity-inconsistent adaptations and health outcomes, which led the team to reevaluate what was considered core fidelity components of the intervention. This example of a cross-sectional study is based on a study by Aschbrenner and colleagues.^34^ | |
| **Cohort Studies.** Cohort studies are used to determine incidence, causes, and prognosis. A cohort is defined as a group of people who share a common characteristic (e.g., smokers, enrollees in a health insurance plan, city residents exposed to synthetic chemicals). Cohort studies may be prospective or retrospective.^35^ | - Prospective cohort studies can be used to track exposure to adaptations and estimate the effect on outcomes. - If the data is available, retrospective cohort studies can be used to perform post-hoc analysis on exposure to adaptations and effect on outcomes. - Cohort studies allow researchers to calculate the effect of each adaptation variable (e.g., number, type, fidelity-consistent or fidelity- inconsistent), significance (major or minor) on the probability of developing the outcome of interest. | - Low incidence of exposure to adaptations will make it difficult to determine effect on outcomes. - When retrospective cohort studies were originally constructed for another purpose, data on all the relevant information on adaptations may be limited. | | **Intervention:** Researchers developed an approach to tracking the impact of planned and unplanned adaptations during the implementation of a population-based health screening program with a large cohort of patients. Adaptations were documented in real time and the data was used in regression models to examine the impact of adaptations on implementation outcomes. This example of a cohort study is based on a study by Allen and colleagues.^36^ | |
| **Case-Control Studies.** Case control studies compare groups retrospectively. Cases with the outcome of interest are matched with a control group who do not have the outcome of interest. Researchers seek to identify possible predictors of outcomes which can generate hypotheses that can be studied via prospective cohort studies and other designs.^35^ | - Case control studies can determine the relative importance of adaptations as predictor variables in relation to the absence or presence of a clinical or implementation outcome. - Case control studies can also be used to examine hypothesized predictor variables of exposure to adaptations or not (adaptations as the outcome). - Useful for hypothesis generation. | - Can only examine one outcome for a given study. - The cases with the outcome may be a biased sample or the controls may be biased (e.g., more innovative hospitals adapt interventions). | | **Intervention:** Researchers conducted a retrospective effectiveness study with matched controls to compare the adapted intervention outcomes against matched cases exposed to a different intervention. Propensity score matching was used to enable case control matching of participants based on available characteristics. The researchers compared clinical outcomes between participants exposed to the adapted intervention versus a different treatment model. There were no significant differences between the two interventions. This example of an adaptive RCT is based on a study by Wakefield and colleagues.^37^ | |
| **Qualitative and Mixed Methods Studies:** *Qualitative research involves using approaches other than quantitative measurement or statistical analysis to gain in depth understanding of concepts, opinions, experiences, and behaviors.^38^ Mixed methods research involves collecting and analyzing qualitative and quantitative data, integrating findings, and drawing inferences in a single study for the purpose of breadth and depth of understanding*.^39^ | | | | | |
| **Qualitative Methods**. Qualitative research involves using approaches other than quantitative measurement or statistical analysis to gain an in depth understanding of concepts, opinions, experiences, and behaviors.^38^ Qualitative methods in implementation research commonly include individual interviews and focus groups, participant observation, and ethnography.^40^ | - Adaptations to interventions or strategies can be examined in depth, including exploring the complexities of and rationale for unplanned adaptations. - Provides a rigorous way to answer “how” and “why” questions in adaptation of interventions or strategies. - Critical for documenting the context in which adaptations occur and the processes of adaptations.   . | | - Generalizability may be limited due to small sample sizes that focus on depth rather than breadth. - Data collection and data analysis (e.g., categorization, coding) can be a labor intensive process. - Confirmation bias is a risk in qualitative research when a study team interprets the data in a biased way that support their hypothesis. - Some health professionals may not want to participate in an in depth discussion of adaptations that drift from fidelity out of concern doing so will impact employment. | | **Intervention/Strategy:** Researchers conducted a qualitative investigation to identify adaptations to the delivery of lung cancer screening interventions. The research team applied the FRAME to probe process adaptations during interviews with lung cancer screening navigators. The research team members conducted a content analysis of the qualitative data guided by the FRAME. The findings identified navigator time and resource considerations for sustainability and scalability of future lung cancer screening interventions. This example of qualitative methods is based on a study by Strayer and colleagues.^41^ |
| **Mixed Methods Research**. Mixed methods research involves collecting and analyzing qualitative and quantitative data, integrating findings, and drawing inferences in a single study for the purpose of breadth and depth of understanding.^39^ Integration is essential to mixed methods and may occur during the design and data collection phase in addition to data analysis and interpretation phases.^42^ | - Sequential explanatory mixed methods design where qualitative data are collected and analyzed to help explain the quantitative results are well suited to investigate the reasons for unplanned adaptations. - Convergent designs (triangulation) where one type of data is used to validate or confirm conclusions reached from analysis of the other type of data are useful for distinguishing between adaptations to the form vs. function of an intervention or strategy. | | - Integrated mixed methods studies can be costly and time consuming, particularly with sequential designs. - Requires specific expertise in mixed methods on the research team - Quantitative and qualitative data can be difficult to integrate and interpret. | | **Intervention/Strategy:** Researchers conducted a mixed methods study to gain knowledge about the context-specific adaptation of a digital health intervention. The research team collected quantitative data using surveys with clinicians and patients while also conducting interviews with patients and focus groups with clinicians to understand how contextual factors influenced experiences with an intervention that was later adapted along with tailoring of its implementation strategies to better fit the context. This example of mixed methods used to investigate context and inform adaptations is based on a study by Valenta and colleagues.^43^ |

**Note:** The examples provided for each study design are informed by the studies cited and may not be exact summaries of the design and/or analytic approach used in the study cited.

**References**

1. Shadish WR, Cook TD, Campbell DT. Experimental and Quasi-Experimental Designs for Generalized Causal Inference. 2001:

2. Cook TD, Campbell DT, Shadish W. *Experimental and quasi-experimental designs for generalized causal inference*. vol 1195. Houghton Mifflin Boston, MA; 2002.

3. Freedland KE, King AC, Ambrosius WT, et al. The selection of comparators for randomized controlled trials of health-related behavioral interventions: recommendations of an NIH expert panel. *J Clin Epidemiol*. Jun 2019;110:74-81. doi:10.1016/j.jclinepi.2019.02.011

4. Rosas LG, Lv N, Xiao L, et al. Effect of a Culturally Adapted Behavioral Intervention for Latino Adults on Weight Loss Over 2 Years: A Randomized Clinical Trial. *JAMA Network Open*. 2020;3(12):e2027744-e2027744. doi:10.1001/jamanetworkopen.2020.27744

5. Nicholls SG, Zwarenstein M, Hey SP, Giraudeau B, Campbell MK, Taljaard M. The importance of decision intent within descriptions of pragmatic trials. *J Clin Epidemiol*. Sep 2020;125:30-37. doi:10.1016/j.jclinepi.2020.04.030

6. Loudon K, Treweek S, Sullivan F, Donnan P, Thorpe KE, Zwarenstein M. The PRECIS-2 tool: designing trials that are fit for purpose. *BMJ : British Medical Journal*. 2015;350:h2147. doi:10.1136/bmj.h2147

7. Zwarenstein M. 'Pragmatic' and 'explanatory' attitudes to randomised trials. *J R Soc Med*. May 2017;110(5):208-218. doi:10.1177/0141076817706303

8. Gold R, Hollombe C, Bunce A, et al. Study protocol for “Study of Practices Enabling Implementation and Adaptation in the Safety Net (SPREAD-NET)”: a pragmatic trial comparing implementation strategies. *Implementation Science*. 2015/10/16 2015;10(1):144. doi:10.1186/s13012-015-0333-y

9. Giraudeau B, Weijer C, Eldridge SM, Hemming K, Taljaard M. Why and when should we cluster randomize? *Journal of Epidemiology and Population Health*. 2024/02/01/ 2024;72(1):202197. doi:<https://doi.org/10.1016/j.jeph.2024.202197>

10. Hemming K, Taljaard M. Key considerations for designing, conducting and analysing a cluster randomized trial. *International Journal of Epidemiology*. 2023;52(5):1648-1658. doi:10.1093/ije/dyad064

11. Bartels SJ, Aschbrenner KA, Pratt SI, et al. Virtual Learning Collaborative Compared to Technical Assistance as a Strategy for Implementing Health Promotion in Routine Mental Health Settings: A Hybrid Type 3 Cluster Randomized Trial. *Adm Policy Ment Health*. Nov 2022;49(6):1031-1046. doi:10.1007/s10488-022-01215-0

12. Hemming K, Haines TP, Chilton PJ, Girling AJ, Lilford RJ. The stepped wedge cluster randomised trial: rationale, design, analysis, and reporting. *BMJ : British Medical Journal*. 2015;350:h391. doi:10.1136/bmj.h391

13. Palmer VJ, Chondros P, Furler J, et al. The CORE study-An adapted mental health experience codesign intervention to improve psychosocial recovery for people with severe mental illness: A stepped wedge cluster randomized-controlled trial. *Health Expect*. Dec 2021;24(6):1948-1961. doi:10.1111/hex.13334

14. Smith J, Brown C. The roll-out implementation optimization (ROIO) design: rigorous testing of a data-driven implementation improvement aim. 2020:

15. Curran GM, Smith JD, Landsverik J, et al. Design and Analysis in Dissemination and Implementation Research. In: Brownson RC, Colditz GA, Proctor EK, eds. *Dissemination and Implementation Research in Health*. Oxford University Press; 2023.

16. Stadnick NA, Laurent LC, Cain KL, et al. Community-engaged optimization of COVID-19 rapid evaluation and testing experiences: roll-out implementation optimization trial. *Implementation Science*. 2023/10/02 2023;18(1):46. doi:10.1186/s13012-023-01306-y

17. Wason JM, Trippa L. A comparison of Bayesian adaptive randomization and multi‐stage designs for multi‐arm clinical trials. *Statistics in medicine*. 2014;33(13):2206-2221.

18. Lauffenburger JC, Choudhry NK, Russo M, Glynn RJ, Ventz S, Trippa L. Designing and conducting adaptive trials to evaluate interventions in health services and implementation research: practical considerations. *BMJ Medicine*. 2022;1(1):e000158. doi:10.1136/bmjmed-2022-000158

19. Davis JM, Masclans L, Rose JE. Adaptive Smoking Cessation Using Precessation Varenicline or Nicotine Patch: A Randomized Clinical Trial. *JAMA Network Open*. 2023;6(9):e2332214-e2332214. doi:10.1001/jamanetworkopen.2023.32214

20. Collins LM, Murphy SA, Strecher V. The multiphase optimization strategy (MOST) and the sequential multiple assignment randomized trial (SMART): new methods for more potent eHealth interventions. *Am J Prev Med*. May 2007;32(5 Suppl):S112-8. doi:10.1016/j.amepre.2007.01.022

21. Lei H, Nahum-Shani I, Lynch K, Oslin D, Murphy SA. A "SMART" design for building individualized treatment sequences. *Annu Rev Clin Psychol*. 2012;8:21-48. doi:10.1146/annurev-clinpsy-032511-143152

22. Almirall D, Compton SN, Gunlicks-Stoessel M, Duan N, Murphy SA. Designing a pilot sequential multiple assignment randomized trial for developing an adaptive treatment strategy. *Stat Med*. Jul 30 2012;31(17):1887-902. doi:10.1002/sim.4512

23. Kilbourne AM, Almirall D, Eisenberg D, et al. Protocol: Adaptive Implementation of Effective Programs Trial (ADEPT): cluster randomized SMART trial comparing a standard versus enhanced implementation strategy to improve outcomes of a mood disorders program. *Implementation Science*. 2014/09/30 2014;9(1):132. doi:10.1186/s13012-014-0132-x

24. Collins LM, Murphy SA, Nair VN, Strecher VJ. A strategy for optimizing and evaluating behavioral interventions. *Annals of Behavioral Medicine*. 2005;30(1):65-73.

25. Broder-Fingert S, Kuhn J, Sheldrick RC, et al. Using the Multiphase Optimization Strategy (MOST) framework to test intervention delivery strategies: a study protocol. *Trials*. 2019/12/16 2019;20(1):728. doi:10.1186/s13063-019-3853-y

26. Bernal JL, Cummins S, Gasparrini A. Interrupted time series regression for the evaluation of public health interventions: a tutorial. *Int J Epidemiol*. Feb 1 2017;46(1):348-355. doi:10.1093/ije/dyw098

27. Bloom HS. Using "short" interrupted time-series analysis to measure the impacts of whole-school reforms. With applications to a study of accelerated schools. *Eval Rev*. Feb 2003;27(1):3-49. doi:10.1177/0193841x02239017

28. Craig P, Katikireddi SV, Leyland A, Popham F. Natural Experiments: An Overview of Methods, Approaches, and Contributions to Public Health Intervention Research. *Annual Review of Public Health*. 2017;38(Volume 38, 2017):39-56. doi:<https://doi.org/10.1146/annurev-publhealth-031816-044327>

29. Madsen KA. School-Based Body Mass Index Screening and Parent Notification: A Statewide Natural Experiment. *Archives of Pediatrics & Adolescent Medicine*. 2011;165(11):987-992. doi:10.1001/archpediatrics.2011.127

30. Cook TD, Campbell DT. The design and conduct of true experiments and quasi-experiments in field settings. *Reproduced in part in Research in Organizations: Issues and Controversies*. Goodyear Publishing Company; 1979.

31. Wu E, Lee YG, Vinogradov V, et al. Intervention Adaptation and Implementation Method for Real-World Constraints and Using New Technologies. *Res Soc Work Pract*. Jul 2023;33(5):562-570. doi:10.1177/10497315221120605

32. Chacón-Moscoso S, Shadish W. Observational studies and quasi-experimental designs: Similarities, differences and generalizations. *Metodologia de las Ciencias del Comportamiento*. 2001;3(2):283-290.

33. Wang X, Cheng Z. Cross-Sectional Studies: Strengths, Weaknesses, and Recommendations. *Chest*. 2020/07/01/ 2020;158(1, Supplement):S65-S71. doi:<https://doi.org/10.1016/j.chest.2020.03.012>

34. Aschbrenner KA, Bond GR, Pratt SI, et al. Evaluating agency-led adaptions to an evidence-based lifestyle intervention for adults with serious mental illness. *Implementation Research and Practice*. 2020/01/01 2020;1:2633489520943200. doi:10.1177/2633489520943200

35. Mann CJ. Observational research methods. Research design II: cohort, cross sectional, and case-control studies. *Emergency Medicine Journal*. 2003;20(1):54-60. doi:10.1136/emj.20.1.54

36. Allen CG, Judge DP, Nietert PJ, et al. Anticipating adaptation: tracking the impact of planned and unplanned adaptations during the implementation of a complex population-based genomic screening program. *Transl Behav Med*. Jun 9 2023;13(6):381-387. doi:10.1093/tbm/ibad006

37. Wakefield S, Delgadillo J, Kellett S, White S, Hepple J. The effectiveness of brief cognitive analytic therapy for anxiety and depression: A quasi-experimental case—control study. *British Journal of Clinical Psychology*. 2021;60(2):194-211. doi:<https://doi.org/10.1111/bjc.12278>

38. Strauss A, Corbin JM. *Basics of qualitative research: Grounded theory procedures and techniques*. Basics of qualitative research: Grounded theory procedures and techniques. Sage Publications, Inc; 1990:270-270.

39. Creswell JW, Clark VLP. *Designing and Conducting Mixed Methods Research*. SAGE Publications; 2011.

40. Hamilton AB, Finley EP. Qualitative methods in implementation research: An introduction. *Psychiatry Res*. Oct 2019;280:112516. doi:10.1016/j.psychres.2019.112516

41. Strayer TE, Spalluto LB, Burns A, et al. Using the Framework for Reporting Adaptations and Modifications-Expanded (FRAME) to study adaptations in lung cancer screening delivery in the Veterans Health Administration: a cohort study. *Implement Sci Commun*. Jan 12 2023;4(1):5. doi:10.1186/s43058-022-00388-x

42. Palinkas LA, Mendon SJ, Hamilton AB. Innovations in Mixed Methods Evaluations. *Annu Rev Public Health*. Apr 1 2019;40:423-442. doi:10.1146/annurev-publhealth-040218-044215

43. Valenta S, Ribaut J, Leppla L, et al. Context-specific adaptation of an eHealth-facilitated, integrated care model and tailoring its implementation strategies—A mixed-methods study as a part of the SMILe implementation science project. Original Research. *Frontiers in Health Services*. 2023-February-17 2023;2doi:10.3389/frhs.2022.977564
